# Genome-wide identification of microRNAs responsive to *Ectropis oblique* feeding in tea plant (*Camellia sinensis* L.)

**DOI:** 10.1038/s41598-017-13692-7

**Published:** 2017-10-19

**Authors:** Anburaj Jeyaraj, Shengrui Liu, Xiao Zhang, Ran Zhang, Mingzhu Shangguan, Chaoling Wei

**Affiliations:** State Key Laboratory of Tea Plant Biology and Utilization, Anhui Agricultural University, 130 Changjiang West Road, Hefei, 230036 P.R. China

## Abstract

The tea plant (*Camellia sinensis* L.) is vulnerable to the geometrid *Ectropis oblique*; although microRNAs (miRNAs) are important for plant growth, development and stress response, the function of miRNAs in the response of *C*. *sinensis* to stress from *E*. *oblique* is unclear. To identify *E*. *oblique* stress-responsive miRNAs and their target genes in tea plant, three small RNA libraries were constructed from leaves subjected to mechanical wounding (MW), geometrid attack (GA) and from healthy control (CK) leaves. Using high-throughput sequencing, 130 known miRNAs and 512 novel miRNAs were identified; of these, differential expression under GA stress was observed for 36 known and 139 novel miRNAs. Furthermore, 169 GA-responsive and 173 MW-responsive miRNAs were detected by miRNA microarray. The expression patterns of six GA-responsive miRNAs were validated by qRT-PCR. Several target genes for these miRNAs encode various transcription factors, including ethylene-responsive transcription factors and squamosa promoter-binding-like proteins, which suggests that these miRNAs may regulate stress-responsive transcriptional processes in tea plant. The present findings provide novel insights into miRNA-mediated regulatory mechanisms underlying the response to GA stress, and also offer valuable information for development of pest resistance using RNA interference-based strategies in tea plants.

## Introduction

The study of plant-herbivore interactions is one of the most important and multidisciplinary undertakings in plant biology research. The evolutionary race between plants and insects has resulted in the development of an elegant defense system in plants, which is capable of recognizing foreign molecules or signals from damaged cells and activating a plant immune response against herbivores^1,2^. Plants respond to insect-induced stress via both direct and indirect defense mechanisms. Direct defense mechanisms refer to plant characteristics that provide mechanical protection such as thicker leaves, hairs, trichomes, thorns and spines; direct defense mechanisms also include production of toxic chemicals that kill or retard the development of herbivores such as terpenoids, alkaloids, anthocyanins, phenols and quinines^3^. Indirect defenses refer to the release of a blend of volatile substances that attract natural predators of the herbivores by providing food (extra floral nectar) and shelter to predators^4^. Both defense actions are mediated through various biochemical, morphological and molecular mechanisms. Even though many biochemical compounds, morphological changes and gene regulations have been found to play roles in plant-insect defense responses, the mechanism by which plants protect themselves from insects is still unclear. Recent evidence shows that small regulatory RNAs have important functions in plant stress responses by targeting multiple transcription factors, which in turn, regulate the expression of various downstream genes involved in the stress response^5^.

Small non-protein-coding RNAs (sRNAs) have emerged as key eukaryotic regulatory molecules, and are involved in diverse biological processes including transcription, oxidation-reduction, transport, and stress response. The majority of sRNAs in plants are 20–24 nucleotides in length, and they are classified into two major classes based on their biogenesis: microRNA (miRNA) is derived from single-stranded stem-loop precursor structure, and small interfering RNA (siRNA) is processed from perfect double-stranded RNA^6–8^. Plant miRNAs play important roles in target gene regulation at the transcriptional and/or post-transcriptional level through perfect or near-perfect complementarities^6^. Recently, miRNAs were reported to play central roles in important plant functions such as regulation of cell growth and development, biotic and abiotic stress response, and gene translational repression^9,10^. For example, ath-miR156 regulates the transition from the juvenile to adult phase by targeting specific transcription factors for silencing in *Arabidopsis thaliana* ^11^.

Over the past decade, numerous conserved and novel miRNAs have been identified through computational and experimental approaches in diverse plant species^9,12^. Recently, high-throughput sequencing technologies have been widely used for the identification of conserved and less abundant novel miRNAs in several plant species, such as *Dendrocalamus latiflorus* ^13^, *Populus euphratica* ^14^ and *Zea mays* ^15^. High-throughput sequencing is useful for the identification of miRNAs in various plant species whose genomes are not yet available in public databases^16^. To date, 28,645 precursor miRNAs and 35,828 mature miRNAs have been deposited in the miRNA database miRBase (http://www.mirbase.org, release 21)^17^.

The tea plant (*Camellia sinensis* (L.) O. Kuntze), is an economically important evergreen woody perennial plantation crop, which is mainly cultivated in tropical and sub-tropical climates. Tea plant is often exposed to biotic stresses from high insect populations; the tea geometrid (*Ectropis oblique*, a chewing insect) is one of the most common pests of tea in China^18^. *E*. *oblique* larvae feed on tea leaves and tender buds during the growing season, and cause considerable mechanical damage to the green tissues of leaves. A severe infestation of tea plants can lead to complete defoliation, greatly reducing tea production^19,20^. Therefore, it is important from an economical perspective to understand the stress response mechanisms of tea plants to *E*. *oblique* feeding and infestation.

Recent studies have identified several tea miRNAs by computational analysis of expressed sequence tags (ESTs)^21–23^. Mohanpuria and Yadav^24^ isolated and cloned six novel miRNAs via the direct cloning approach, and Jeyaraj *et al*.^25^ analyzed the expression of 15 tea miRNAs during bud dormancy using stem-loop pulse RT-qPCR. Most recently, several conserved and novel miRNAs were identified in response to drought and cold stress in the tea plant by high-throughput sequencing^26–28^. However, the functions and mechanisms by which these miRNAs affect the stress response in *C*. *sinensis* are unclear. Studies on the effect of mechanical and geometrid-induced stress on tea plant miRNAs may improve our understanding of miRNA functions in response to biotic stress. This study aims to identify the differential conserved and species-specific novel miRNAs responsive to insect feeding in tea plant by high-throughput small RNA sequencing; to achieve this, we constructed three small RNA (sRNA) libraries from tea plant leaves subjected to mechanical wounding (MW), geometrid attack (GA), and healthy control (CK) leaves. A total of 130 conserved and 512 novel miRNAs were identified among the libraries. The potential target genes of these miRNAs were involved in diverse biological processes including transcription, signal transduction, stress response, and plant growth and maintenance. These results lay the foundation for understanding miRNA-based regulation in the response to the infestation of tea plants with *E*. *oblique*.

## Results

### Sequence analysis of sRNAs

**Figure 1:**
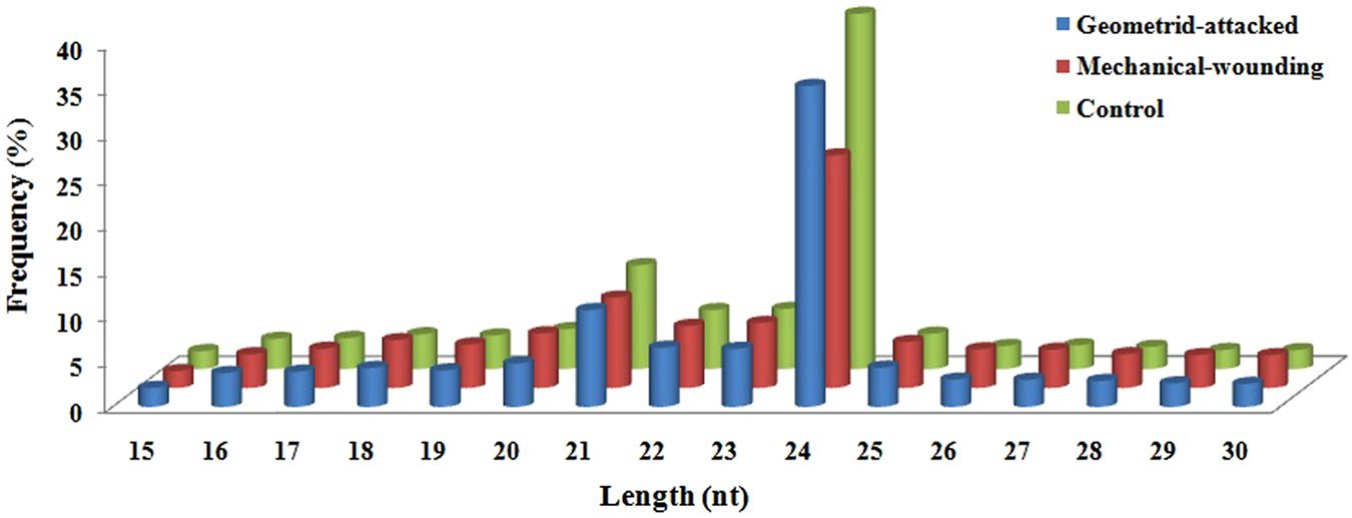
Size distribution and abundances of small RNAs in the libraries of the geometrid-attacked, mechanical-wounding and control samples of *Camellia sinensis*.

In order to identify conserved and novel miRNAs in tea, three different small RNA libraries were constructed. Each sRNA library was generated from an equal mixture of small RNAs obtained at different time points from GA, MW and CK tea leaves, respectively. The three sRNA libraries were sequenced by Solexa sequencing technology using an Illumina GAIIX system provided by LC Sciences (Houston, Texas, USA). A total of 9,323,041, 11,910,328 and 14,320,517 raw reads were generated from the three sRNA libraries, respectively (Table 1). To evaluate the efficiency of Solexa sequencing and the quality of the sequences, all reads were annotated and classified by aligning them against the miRBase 21 database (http://www.mirbase.org/), the Rfam database (http://rfam.janelia.org) and the Repbase database (http://www.girinst.org/repbase). Thereafter, sRNAs were classified into different categories according to their annotations as 3′ adapter (ADT) and length filter, junk reads, Rfam, mRNA, repeats, rRNA, tRNA, snRNA and snoRNA sequences and other Rfam RNA sequences (Table 1). We obtained 4,315,700 clean reads that represented 2,363,062 unique sequences from the GA library, 5,057,553 clean reads that represented 2,207,927 unique sequences from the MW library, and 7,151,289 clean reads that represented 3,775,038 unique sequences from the CK library (Table 1). The length distribution of the sRNAs ranged from 15 to 30 nt, with 24-nt sRNAs representing the most frequent length in the three libraries (Table 2 and Fig. 1). The percentage of 24-nt sRNAs in the CK library (39.13%) was notably higher than that in both the GA (35.36%) and MW (25.61%) library (Table 2). The 21-nt sRNA sequences represented the next most abundant class, which accounted for 11.44%, 10.66% and 9.95% of the reads in the CK, GA and MW libraries, respectively. The next most abundant class was the 23-nt sRNA sequences, which represented 6.62%, 6.39% and 7.16% of the CK, GA and MW libraries, respectively. It was followed by the 22-nt class, which represented 6.49%, 6.53% and 6.83% of the CK, GA, and MW libraries, respectively (Table 2).

**Table 1:** Overview of reads from the raw data to cleaned sequences from small RNA libraries.

	GA^*^		MW^*^		CK^*^	
	Total (%)	Unique (%)	Total (%)	Unique (%)	Total (%)	Unique (%)
Raw reads	9323041 (100)	3763136 (100)	11910328 (100)	3752258 (100)	14320517 (100)	5376088 (100)
3′ADT&length filter	2631369 (28.22)	848952 (22.56)	3769752 (31.65)	988872 (26.35)	3445548 (24.06)	963153 (17.92)
Junk reads	29144 (0.31)	24368 (0.65)	26897 (0.23)	20778 (0.55)	37873 (0.26)	31843 (0.59)
Rfam	1017420 (10.91)	247775 (6.58)	1379642 (11.58)	251479 (6.70)	1560662 (10.90)	282995 (5.26)
Repeats	170506 (1.83)	30006 (0.80)	178485 (1.50)	30623 (0.82)	299638 (2.09)	38809 (0.72)
Clean reads	4315700 (46.29)	2363062 (62.80)	5057553 (42.46)	2207927 (58.84)	7151289 (49.94)	3775038 (70.22)
rRNA	485398 (5.21)	134102 (3.56)	646150 (5.43)	133552 (3.56)	726837 (5.08)	148924 (2.77)
tRNA	270471 (2.90)	64748 (1.72)	474458 (3.98)	67185 (1.79)	409233 (2.86)	72505 (1.35)
snoRNA	66279 (0.71)	11019 (0.29)	56587 (0.48)	11480 (0.31)	115028 (0.80)	14202 (0.26)
snRNA	25631 (0.27)	9272 (0.25)	45625 (0.38)	10249 (0.27)	43670 (0.30)	11900 (0.22)
other Rfam RNA	169641 (1.82)	28634 (0.76)	156822 (1.32)	29013 (0.77)	265894 (1.86)	35464 (0.66)
miRNA	141482 (1.52)	1198 (0.03)	118357 (0.99)	1100 (0.03)	264845 (1.85)	1255 (0.02)

*GA: geometrid-attacked, MW: mechanical-wounding, CK: control.

**Table 2:** Length distribution and abundances of small RNAs in the libraries of geometrid-attacked, mechanical-wounding and control samples from *Camellia sinensis*.

Length (nt)	GA*		MW*		CK*	
	Total (%)	Unique (%)	Total (%)	Unique (%)	Total (%)	Unique (%)
15	89786 (2.08)	25437 (1.08)	94250 (1.86)	24553 (1.11)	140732 (1.97)	30731 (0.81)
16	161257 (3.74)	51358 (2.17)	187684 (3.71)	52394 (2.37)	237392 (3.32)	64150 (1.70)
17	167392 (3.88)	65565 (2.77)	218734 (4.32)	70348 (3.19)	244718 (3.42)	83433 (2.21)
18	185167 (4.29)	73240 (3.10)	266660 (5.27)	81115 (3.67)	272934 (3.82)	97024 (2.57)
19	173306 (4.02)	77863 (3.30)	242013 (4.79)	85834 (3.89)	264411 (3.70)	108406 (2.87)
20	206004 (4.77)	84808 (3.59)	304324 (6.02)	93487 (4.23)	314686 (4.40)	122963 (3.26)
21	459911 (10.66)	151740 (6.42)	503205 (9.95)	152702 (6.92)	818415 (11.44)	237431 (6.29)
22	281749 (6.53)	137108 (5.80)	345255 (6.83)	137196 (6.21)	464130 (6.49)	213844 (5.66)
23	275840 (6.39)	152819 (6.47)	362095 (7.16)	145437 (6.59)	473450 (6.62)	290761 (7.70)
24	1525950 (35.36)	1191837 (50.44)	1295079 (25.61)	930907 (42.16)	2798386 (39.13)	2081748 (55.15)
25	186871 (4.33)	111056 (4.70)	255795 (5.06)	114308 (5.18)	278848 (3.90)	161533 (4.28)
26	129722 (3.01)	54169 (2.29)	215168 (4.25)	70516 (3.19)	181688 (2.54)	65779 (1.74)
27	128695 (2.98)	50370 (2.13)	212529 (4.2)	65709 (2.98)	186565 (2.61)	58749 (1.56)
28	122941 (2.85)	47275 (2.00)	188586 (3.73)	63101 (2.86)	175112 (2.45)	55528 (1.47)
29	111781 (2.59)	44969 (1.90)	183654 (3.63)	60828 (2.75)	150175 (2.10)	52416 (1.39)
30	109328 (2.53)	43448 (1.84)	182522 (3.61)	59492 (2.69)	149647 (2.09)	50542 (1.34)

*GA: geometrid-attacked, MW: mechanical-wounding, CK: control.

### Identification of conserved miRNAs

**Figure 2:**
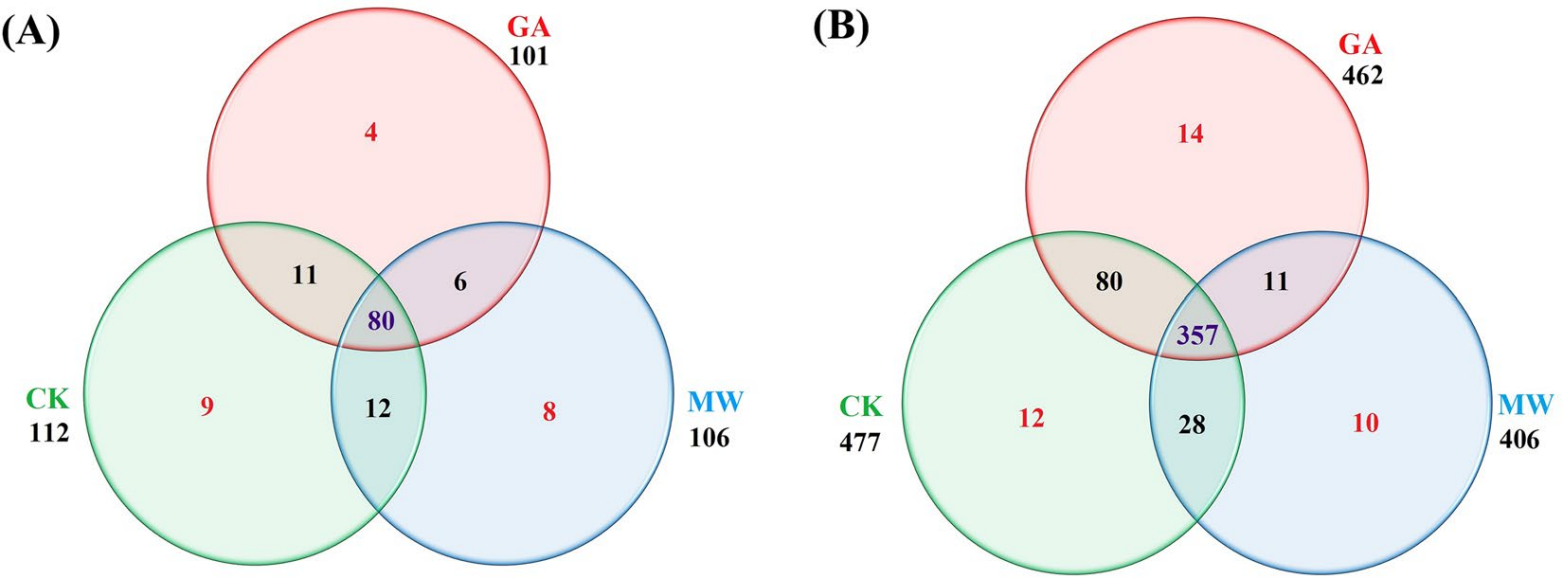
Venn diagrams showing the number of conserved (**A**) and novel (**B**) miRNAs expressed in the geometrid-attacked (GA), mechanical-wounding (MW) and control samples (CK). A: conserved miRNAs, B: novel miRNAs.

**Figure 3:**
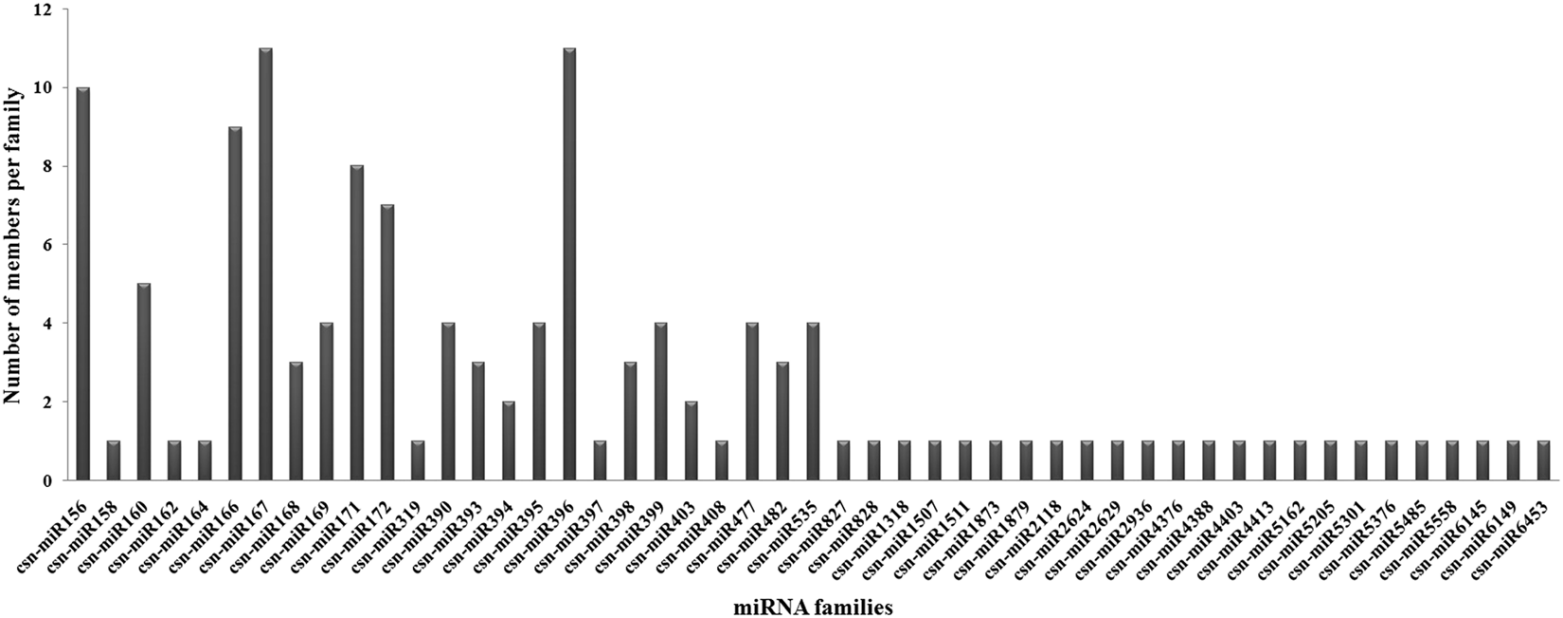
Number of conserved miRNA families in *Camellia sinensis*.

In order to investigate the conserved miRNAs present in tea, the unique sRNA sequences generated from three sRNA libraries (CK, GA and MW) were aligned against known plant miRNAs deposited in miRBase 21.0, allowing a maximum of two mismatches. A total of 130 conserved miRNAs belonging to 49 miRNA families with high sequence matches to currently known plant miRNAs were identified in the three libraries (Supplementary Table S1). Of these, 112 conserved miRNAs were detected in the CK library, and 101 and 106 miRNAs were detected in the GA and MW libraries, respectively (Supplementary Table S1 and Fig. 2A). Among these, six conserved miRNAs (csn-miR166a-3p, csn-miR166c-3p, csn-miR168a, csn-miR396a-5p, csn-miR396d-5p and miR535a) showed a higher number of reads in the CK library than in the GA and MW libraries. Thus, these miRNAs may play a specific role in GA- and MW-associated stress. Moreover, several moderately conserved miRNA families, including miR156, miR160, miR167, miR172 and miR390, had a number of sequence reads that ranged from 100 to 1000 in at least one liabrary. The remaining lowly conserved miRNAs had fewer than 100 reads in the three libraries (Supplementary Table S1). The number of members in the conserved miRNA families differs dramatically across all three libraries. The miRNA families miR156, miR166, miR167, miR171, miR172 and miR396 had more than five members each. Six miRNA families (miR169, miR390, miR395, miR399, miR477 and miR535) had four members each, and the majority of miRNA families had only one member each (Supplementary Table S1 and Fig. 3).

### Identification of novel miRNAs

To identify novel miRNA sequences, all unannotated unique sRNA sequences were mapped to EST sequences and the scaffold sequences that were assembled on the base of the genome survey dataset (unpublished data). The stem-loop structure of miRNA precursors was used to predict novel miRNAs using the Mfold program^29^. According to the recent criteria for annotating novel plant miRNAs based on miRNA:miRNA*^30^, we successfully identified 512 novel miRNAs in all three libraries (Supplementary Table S2). Of these, 477 novel miRNAs were detected in the CK library, whereas 462 and 406 novel miRNAs were detected in the GA and MW libraries, respectively. These findings indicate that the expressions of certain novel miRNAs are specifically repressed by GA- and MW-induced stress (Supplementary Table S2 and Fig. 2B). The novel miRNAs sequences were between 18 and 25 nt in length, and the 24 nt reads were the most abundant. The lengths of novel miRNA precursors ranged from 57 to 219 nt, with an average length of 148 nt. The negative folding free energies of the novel miRNA precursors ranged from −164.9 to −18.2 kcal mol^−1^, with an average value of −74.62 kcal mol^−1^. The stem-loop structures of these predicted novel miRNA precursor sequences were shown in Supplementary Table S2.

### Differential expression analysis of miRNAs

To identify the miRNAs involved in the response to mechanical and *E*. *oblique*-induced stresses, expression levels of all conserved and novel miRNAs in the three libraries were normalized, and the differential expression levels were estimated from the read counts obtained from high-throughput sequencing. In the three libraries, miRNAs with higher than two-fold change in their expression (log2 value) (GA vs. CK, MW vs. CK, and GA vs. MW) were considered to be up-regulated, while miRNAs with less than −0.5-fold change in their expression (log2 value) (GA vs. CK, MW vs. CK, and GA vs. MW) were considered to be down-regulated.

**Figure 4:**
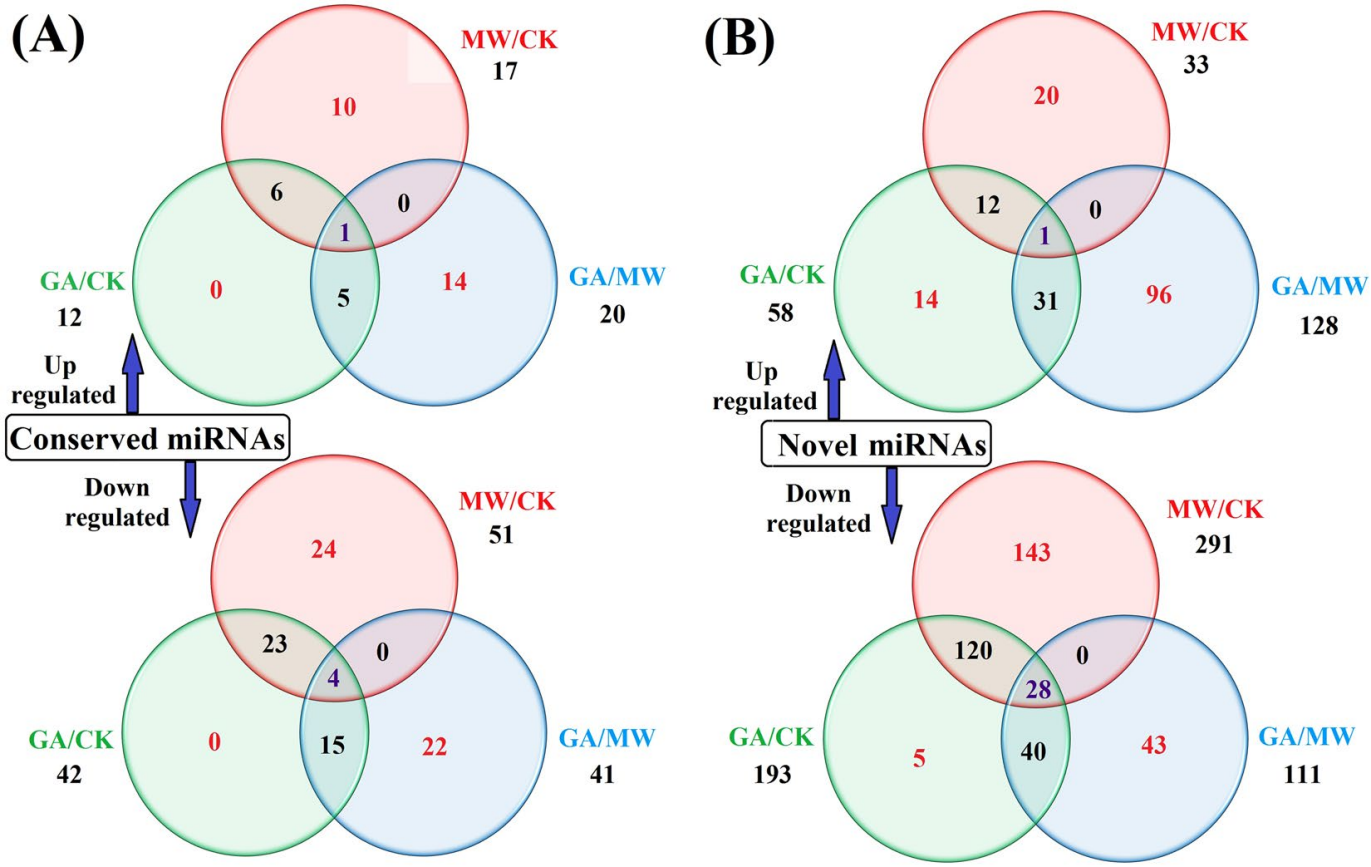
Venn diagrams showing the differentially expressed conserved (**A**) and novel (**B**) miRNAs under the geometrid-attack (GA), mechanical-wounding (MW) and control (CK) conditions. The number of miRNAs in each category is provided in each region.

In GA/CK comparison, a total of 54 conserved miRNAs were differentially expressed, including 12 up-regulated and 42 down-regulated miRNAs; 251 novel miRNAs showed significant differential expression, of which 58 and 193 miRNAs were up-regulated and down-regulated, respectively (Fig. 4). Thus, differential expression of these conserved and novel miRNAs could have been induced by either MW or oral secretion (OS) stimulation or both.

To identify the MW-induced miRNAs, we performed a comparison between the MW and CK libraries. Accordingly, we identified 68 conserved miRNAs that were differentially expressed, of which 17 and 51 miRNAs were up-regulated and down-regulated, respectively. Moreover, 324 novel miRNAs were expressed differentially between the two libraries, including 33 up-regulated and 291 down-regulated miRNAs.

To identify miRNAs specifically induced by OS of *E*. *oblique*, we conducted a comparison between the GA and MW libraries. A total of 61 conserved and 239 potentially novel miRNAs were identified as differentially expressed; of these 148 miRNAs (20 conserved and 128 novel miRNAs) were up-regulated and 152 miRNAs (41 conserved and 111 novel miRNAs) were down-regulated (Fig. 4). Altogether, 5 conserved miRNAs (1 up-regulated and 4 down-regulated) and 29 novel miRNAs (1 up-regulated and 28 down-regulated) showed significant differential expression among GA/CK, MW/CK and GA/MW comparisons (Fig. 4A,B). Many unique conserved and novel miRNAs were identified individually in the three comparisons. In particular, we found that 29 conserved miRNAs (6 up-regulated and 23 down-regulated miRNAs) and 132 novel miRNAs (12 up-regulated and 120 down-regulated miRNAs) were differentially expressed both in the GA/CK and MW/CK comparisons (Fig. 4A,B). In the GA/MW comparison, 1 conserved and 5 novel miRNAs (csn-miR827a, csn-miRn80, csn-miRn83, csn-miRn146, csn-miRn190 and csn-miRn239) showed the most significant upregulation (with fold change as high as 9.20), while two novel miRNAs (csn-miRn51 and csn-miRn152) were the most significantly down-regulated miRNAs (with fold change as low as –8.88). The majority of the differentially expressed miRNAs (e.g. csn-miR167b, csn-miR169b, csn-miR827a, csn-miR1318, csn-miR156h, csn-miR166a-5p, csn-miR172d and csn-miR395g) exhibited significant differences in their expression levels. For instance, 4 conserved and 14 novel miRNAs were only identified in the GA library (Fig. 2A,B); thus, these differentially expressed miRNAs may play critical roles in the response to OS-induced stress in tea plant.

### Microarray analysis of miRNAs

In this study, microarray-based hybridization was used to investigate the expression patterns of miRNAs in the tea plant under mechanical stress and *E*. *oblique*-induced stress. The mixed RNA pool microarray consisted of 642 probes that represented all the predicted miRNAs from high-throughput sequencing. The small-molecular-weight RNAs isolated from the CK, GA and MW libraries were used for microarray hybridization. We compared the relative expression of conserved and novel miRNAs between the GA and CK libraries and between the MW and CK libraries based on the expression signal (signal intensity > 30) respectively. miRNAs with more than or equal to one-fold change in their expression levels (GA vs. CK and MW vs. CK) were considered to be up-regulated, while miRNAs with less than one-fold change in their expression (GA vs. CK and MW vs. CK) were considered to be down-regulated.

Microarray data showed that several known and novel miRNAs had significant differential expression levels under GA or MW stress condition. Among the 642 miRNA probes, a total of 169 miRNAs (43 conserved and 126 novel miRNAs) in the comparison between the GA and CK libraries, and 173 miRNAs (46 conserved and 127 novel miRNAs) in the comparison between the MW and CK libraries showed differential expression profiles by microarray analysis; of these, 75 miRNAs (11 conserved and 64 novel miRNAs) were up-regulated and 48 miRNAs (16 conserved and 32 novel miRNAs) were down-regulated in both the GA vs. CK and MW vs. CK comparisons (Supplementary Tables S3 and S4). In the GA stress condition, 16 conserved miRNAs (6 up-regulated and 10 down-regulated) and 30 novel miRNAs (11 up-regulated and 19 down-regulated) only showed significant differential expression in the GA vs. CK (Supplementary Table S3); thus, these miRNAs may be activated or repressed in response to GA-induced stress. In the MW stress condition, 31 miRNAs (11 conserved and 20 novel miRNAs) were only up-regulated, while 19 miRNAs (8 conserved and 11 novel miRNAs) were only down-regulated in the MW vs. CK (Supplementary Table S4); thus, these miRNAs may be specifically involved in regulation of target genes in response to MW-induced stress.

### Prediction of miRNA targets

**Figure 5:**
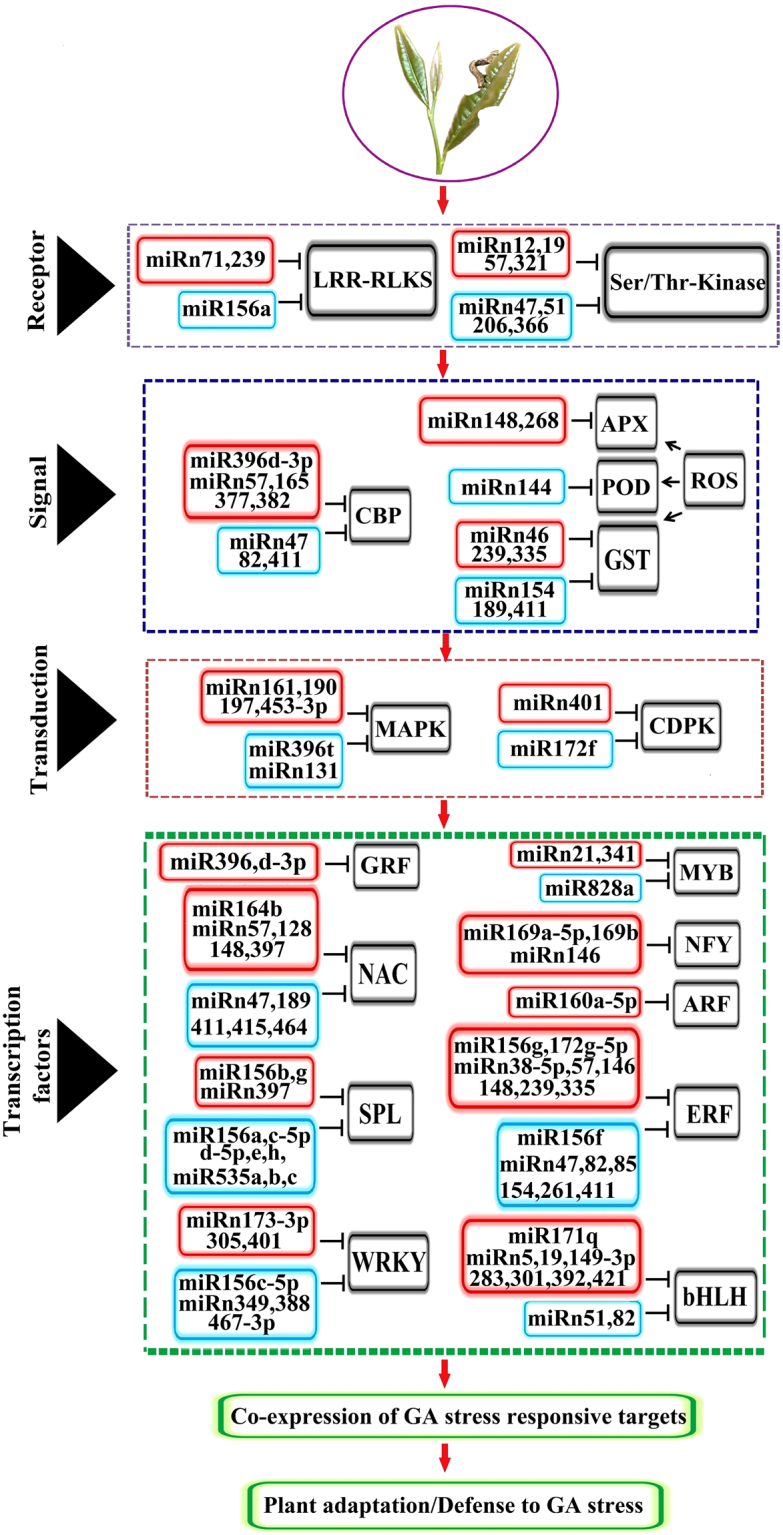
A hypothetical model of GA-responsive miRNA regulatory networks in tea plant. Leucine-rich repeat receptor-like protein kinases (LRR-RLKs), Serine/threonine-protein kinase (Ser/Thr_kinase), Calcium-binding protein (CBP), Reactive oxygen species (ROS), L-ascorbate peroxidase (APX), Peroxidase (POD), Glutathione-S-transferase (GST), Mitogen-activated protein kinase (MAPK), Calcium-binding protein, calcium-dependent protein kinase (CDPK), MYB domain proteins (MYBs), NAC domain transcription factors (NACs), Squamosa promoter-binding-like protein (SPLs), WRKY transcription factor (WRKYs), Nuclear transcription factor Y (NFYs), Auxin response factors (ARFs), Ethylene-responsive transcription factors (ERFs), Basic helix-loop-helix proteins (bHLHs), Growth-regulating factor (GRF). Up- and down-regulated csn-miRNAs are indicated in red and blue boxes respectively. All predicted target genes are represented in black box.

To better understand the biological role of miRNAs in *C*. *sinensis*, we used Target Finder to predict potential target genes of miRNAs based on the transcriptome sequence data of tea plant. Based on their perfect or near-perfect complementarity to their target gene sequences, a total of 1308 potential target genes were identified for 371 miRNAs, including 82 conserved and 289 novel miRNAs (Supplementary Table S5). Several identified target genes for known and novel miRNAs encoded transcription factors; these include key regulators of stress response and plant growth/development genes (Supplementary Table S5) such as squamosa promoter-binding-like protein (SPLs), myb domain proteins (MYBs), auxin response factors (ARFs), ethylene-responsive transcription factors (ERFs), NAC domain transcription factors (NACs), nuclear transcription factor Y (NFYs) and basic helix-loop-helix proteins (bHLHs). Many of the identified target transcripts encoded defense response-related genes, such as leucine-rich repeat receptor-like protein kinases (LRR-RLKs), serine/threonine-protein kinase (Ser/Thr_kinase), calcium-binding protein (CBP), calcium-dependent protein kinase (CDPK) and mitogen-activated protein kinase (MAPK), which are involved in signal sensing and transduction. Some target transcripts were annotated to genes encoding important reactive oxygen species (ROS)-related enzymes, which included peroxidase (POD), l-ascorbate peroxidase (APX) and glutathione-*S*-transferase (GST); these are involved in ROS signaling in herbivory-induced responses in plants. The other predicted miRNA target genes are involved in diverse physiological and metabolic processes, including plant metabolism, transport, cell growth/maintenance and stress responses (Supplementary Table S5). Based on the comprehensive identification of GA-responsive miRNAs and analysis of the predicted target gene functions, we propose a schematic model of the defence mechanism and regulatory networks associated with GA stress response in tea plant (Fig. 5).

### Gene Ontology enrichment and KEGG pathway analysis

**Figure 6:**
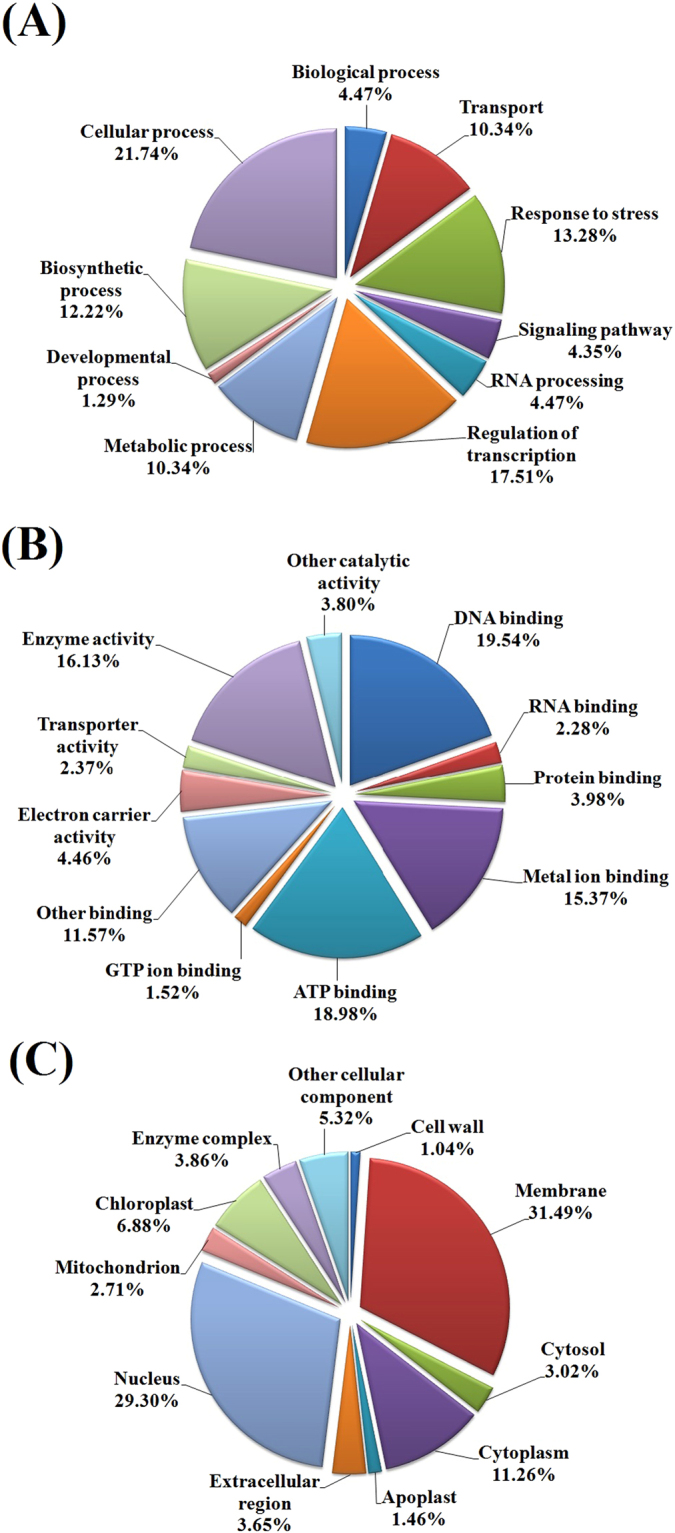
GO enrichment analysis of the predicted potential targets of differentially expressed miRNAs in *Camellia sinensis*. Categorization of the miRNA target genes into biological processes (**A**), molecular functions (**B**) and cellular components (**C**) was performed.

Based on functional annotations, the target genes were classified into three Gene Ontology (GO) categories: biological process, molecular function and cellular component (Fig. 6A, B and C). In the biological process category (Fig. 6A), the five major subcategories were included in cellular processes (21.74%), regulation of transcription (17.51%), response to stresses (13.28%), biosynthetic processes (12.22%), and transport and metabolic processes (10.34%). In the molecular function category (Fig. 6B), the most frequent term was belonged to binding activity (DNA binding and ATP binding); this was followed by enzyme activity, metal ion binding and other binding. With regard to the cellular component category (Fig. 6C), the membrane and the nucleus were the most frequent terms.

**Figure 7:**
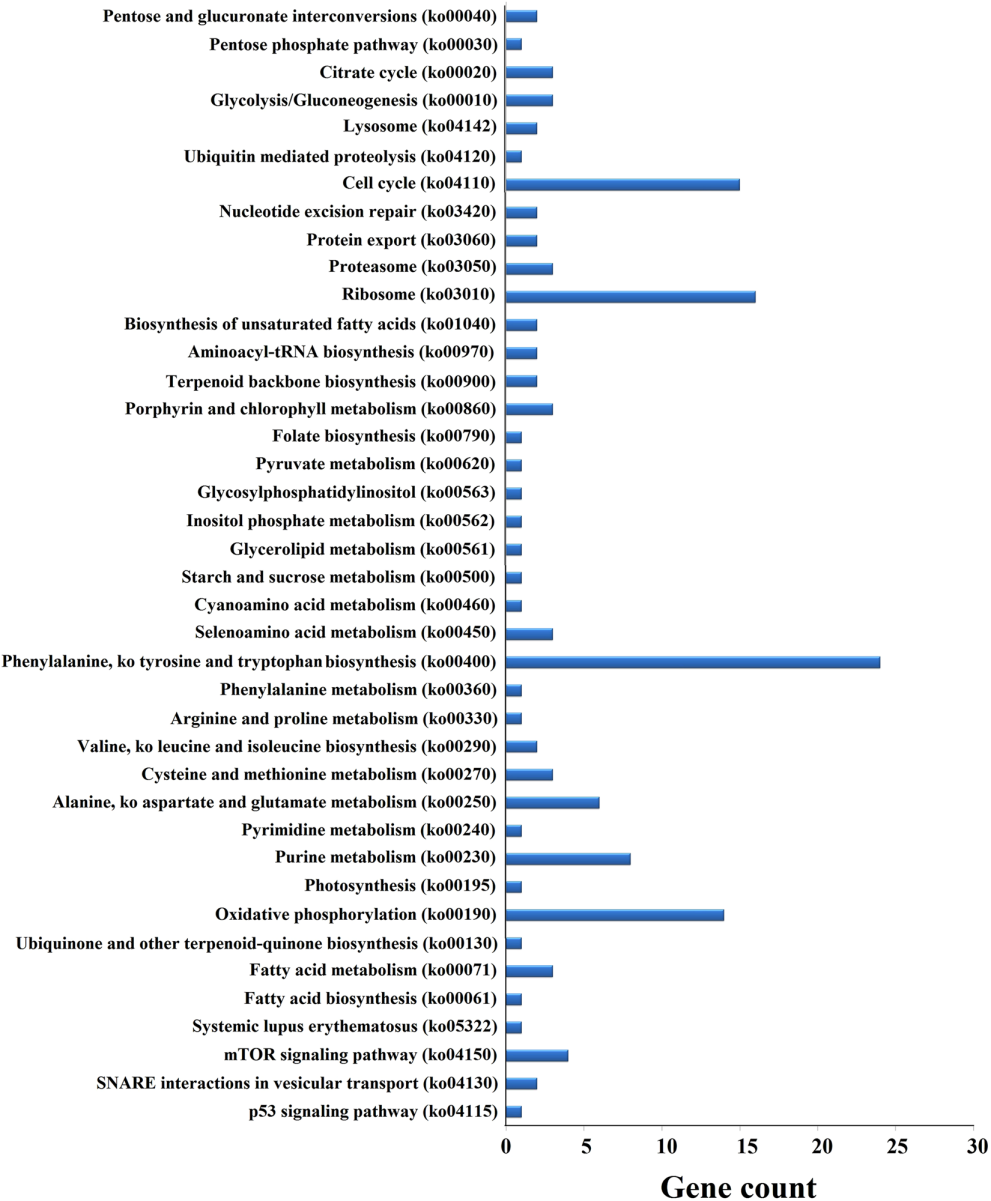
KEGG pathway analysis of the predicted potential targets of differentially expressed miRNAs in *Camellia sinensis*.

Pathway enrichment analysis was based on the KEGG database, and demonstrated that 23 conserved miRNAs and 93 novel miRNAs participated in 30 and 113 metabolic networks, respectively. These networks showed that the miRNA target genes were mainly involved in amino acid and nucleotide metabolism, followed by tyrosine and tryptophan biosynthesis, cell cycle, oxidative phosphorylation and other pathways (Supplementary Table S5 and Fig. 7).

### Validation of miRNA expression with stem-loop qRT-PCR

**Figure 8:**
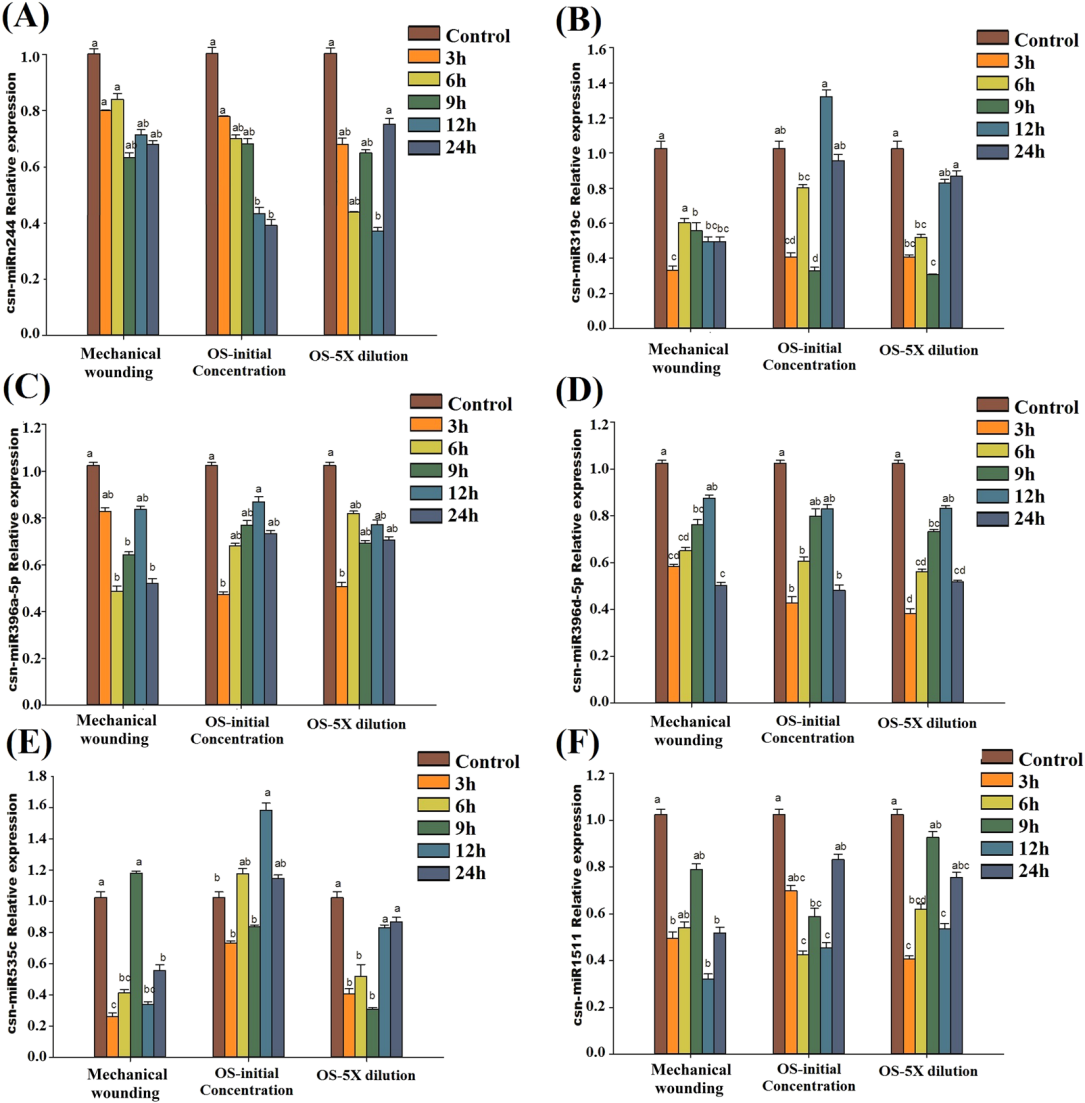
Relative expression levels of six miRNAs in *C*. *sinensis* leaves exposed to mechanical damage and *E*. *oblique* saliva (OS) at different time intervals by qRT-PCR: csnmiRn224 (**A**), csn-miR319c (**B**), csn-miR396a-5p (**C**), csn-miR396d-5p (**D**), csn-miR535c (**E**), and csn-miR1511 (**F**). The expression levels of miRNAs were normalized to that of U6. Relative expression was calculated using the 2^−ΔΔCT^ method. Data represent the means ± SD values (n = 3) of three biological samples. Different letters above bars represent significant differences at *P* < 0.05.

To validate the expression profiles of the miRNAs obtained from the high-throughput sequencing, six GA-responsive miRNAs (csn-miRn244, csn-miR319c, csn-miR396a-5p, csn-miR396d-5p, csn-miR535c and csn-miR1511) were randomly selected for qRT-PCR analysis in tea plant leaves subjected to MW and treated with *E*. *oblique* OS at the initial concentration and OS at 5× dilution at various time intervals (3, 6, 9, 12, and 24 h). As shown in Fig. 8, the analysis of MW-treated tea leaves showed that these six miRNAs were showed lower levels of expression relative to the controls at the following time intervals: csn-miR319c at 3 h, csn-miR396a-5p at 6, 9 and 24 h respectively, csn-miR396d-5p at 3, 6 and 24 h respectively, csn-miR535c and csn-miR1511 at 3, 12 and 24 h respectively. Treatment of tea leaves with OS-initial concentration at 3 h showed that csn-miR319c, csn-miR396a-5p and csn-miR396d-5p had 0.41-, 0.47- and 0.43-fold expression levels, respectively, whereas csn-miRn244 showed 0.39- and 0.50-fold expressions at 12 and 24 h respectively. Similarly, in the OS-5× dilution treatment, four miRNAs showed a significantly decreased expressions at 3 and 6 h respectively: csn-miR319c, csn-miR396d-5p, csn-miR535c and  csn-miR1511. Whereas, the higher expression levels of csn-miR319c and csn-miR535c observed at 12 h with the OS-initial concentration. These results may indicate that these miRNAs play important plant stress response roles in tea by negatively regulating their corresponding target transcripts through translational inhibition or cleavage.

## Discussion

### Identification and pattern of length distribution of miRNAs in tea plant

Plant miRNAs are important regulators that play versatile biological functions such as development, environmental adaptation and stress tolerance. There are several recent studies describing miRNA expression in tea plant subjected to abiotic stresses, including cold and drought stresses^26–28^. However, there are few studies concerning the identification and expression of differential miRNAs under biotic stress, such as that caused by *E*. *oblique* which causes extreme damage to leaves. To understand the expression patterns of miRNAs and their potential biological fucntions in response to insect herbivory in tea plant, we assembled the miRNA expression profiles of the CK, GA, and MW libraries using high-throughput sequencing. As a result, a total of 130 conserved and 512 novel miRNAs were identified in the three libraries. We observed that most conserved and novel tea plant sRNA reads were 24 nt in length, followed by 21 nt, 23 nt and 22 nt reads (Table 2 and Fig. 1). The abundances of sRNA reads between 15 nt and 20 nt were not significant. Similar patterns of length distribution of sRNAs were also reported in several previous studies in tea plant^26–28^ and in several other plant species^31–33^.

### Differentially expressed miRNAs induced by oral secretion of *E*. *oblique*

Plant defense responses against pest attack were induced by two types of stimuli: mechanical wounding and oral secretion^18^. The MW/CK comparison identified 17 up-regulated and 51 down-regulated conserved miRNAs, as well as 33 up-regulated and 291 down-regulated novel miRNAs (Supplementary Tables S1, S2 and Fig. 4). To identify miRNAs solely induced by *E*. *oblique* OS, we evaluated the differential expression profiles of miRNAs in the GA/MW comparison. In total, 14 up-regulated conserved miRNAs belonging to 10 miRNA families and 22 down-regulated conserved miRNAs belonging to 17 miRNA families were identified, respectively (Fig. 4). Moreover, 96 up-regulated and 43 down-regulated novel miRNAs were identified in the GA/MW comparison, indicating that these miRNAs may also play significant roles in response to OS-induced stress. Although some miRNAs were also induced by MW, expression of these miRNAs changed significantly in GA relative to both CK and MW treated leaves; therefore, these miRNAs were also classified as OS-inducible miRNAs.

Previous studies demonstrated that miR156g, miR167, miR172k and miR396 were up-regulated under cold and drought stresses in tea plant^26,28^. In *Arabidopsis*, miR156g, miR167, miR171 and miR396 were found to be up-regulated in response to salt stress^34^. Interestingly, some miRNAs of the same families (i.e. miR156, miR167, miR171, miR172 and miR396) were identifed in both up- and down-regulated groups (Supplementary Table S1); several previous studies had similar observations^35–37^. In this study, members of miR160, miR166, miR319, miR393 and miR2118 families were only down-regulated in the GA/MW comparison, and these miRNAs have been proven to be critial regulators in plant development and various stress responses. It is noteworthy that previous studies showed miR477 and miR395 were up-regulated under GA stress, but there was no consistent regulatory pattern under abiotic stress responses in tea plant^26,28^. Nevertheless, miR477 and miR395 families were found to be up-regulated under cold stress in *Populus* ^35^ and salt stress in maize^38^. This differential expression may reflect distinct miRNA functions in plant-herbivory interactions. In addition to conserved miRNAs, many novel miRNAs were identified as significantly up- or down-regulated in the GA/MW comparison, although little is known about their potential functions in plants. These findings may reflect the functions of different miRNAs in different tea plant-pest interactions, suggesting that these miRNAs have complex regultory functions in tea plant subjected to OS-induced stress.

### OS-inducible miRNAs orchestrate defense response signaling pathways

The defense mechanisms induced by OS of insect herbivory in tea plant can be classified into four main pathways: receptor recognition, signals perception, signal transduction, and transcription factors and defense genes^18^. Based on our results, we prepared a schematic illustration of our hypothetical model for regulatory networks of OS-responsive miRNAs and their target genes in tea plant (Fig. 5). Various elicitors in oral secretions resulting from insect herbivory can enter into plants through wounds, and induce the resistance response in plants. These elicitors can be recognized by plant multiple receptors, such as Leucine-rich repeat receptor-like protein kinase (LRR-RLKs) and serine/threonine (Ser/Thr) protein kinase^18,39,40^, which in turn activate signal transduction pathways that regulate distinct resistance-gene expression systems and induce the plant to produce diverse defense metabolites^41^. Remarkably, our results showed that the LRR-RLKs can be targeted by a conserved csn-miR156a (down-regulated) and two novel miRNAs (up-regulated); in addition, Ser/Thr-kinase can be targeted by six novel miRNAs (three up-regulated and three down-regulated) (Fig. 5). These miRNAs may function by targeting LRR-RLKs and Ser/Thr-kinase receptors, thereby triggering Ca^2+^ influxes and Ca^2+^–binding protein activation through MAPK cascades, followed by a series of processes such as phytohormone and ROS biosynthesis pathways^18,42^.

Studies have shown that Ca^2+^-related proteins are involved in modulating plant defense responses against insect herbivory^18,42^. During the process of signal perception, the conserved csn-miR396d-3p and four novel miRNAs were up-regulated, along with the down-regulation of three novel miRNAs (Fig. 5); these targeted genes encoding calcuim-binding protein (CBP). Liu *et al*.^28^ found that two Ca^2+^-signaling-related kinase genes were targeted by two miRNAs (miR8762d and miR2586a) in tea plant. Our findings indicate that these miRNAs are likely involved in signal transduction under OS-induced stress in *C. sinensis*. ROS production is an essential component of plant stress responses, which are implicated in herbivory-induced responses in plants^39,43^. Evidence also showed that ROS-related enzymes such as peroxidase (POD), superoxide dismutase (SOD), glutathione S-transferases (GST) and ascorbate peroxidase (APX) could cause damage to insect cells and tissues^18,44^. Notably, APX, POD, and GST were identified to be potential targets by 2, 1, and 6 novel miRNAs, respectively, in this study (Fig. 5).

Protein kinases with phosphorylation activity play critial roles in environmental signal perception, transduction and amplification. It is well-established that MAPKs and CDPKs act as critical regulators by modulating multiple defense responses in plant disease resistance^45,46^. Consequently, we observed that MAPKs were targeted by four up-regulated miRNAs and two down-regulated miRNAs (including the conserved miR396t); CDPKs were targeted by one up-regulated miRNA and one down-regulated miRNA (including the conserved miR172f) (Fig. 5). This finding suggests that miRNAs are involved in protein kinase induced signal events, thereby triggering the miRNA-mediated defense responses. Overall, the miRNAs may function by regulating Ca^2+^-related protein genes, protein kinase genes, or ROS-related genes, thereby inducing multiple defense responses against OS-induced stresses in tea plant.

### OS-inducible miRNAs targeting transcription factors involved in defense responses

Transcription factors function as master switches which can control the expression of genes, and regulate various aspects of plant development and responses to biotic and abiotic stresses^47^. It is well known that transcription factors are the predominant targets of miRNAs both in animals and plants^48^. Unsurprisingly, most of *E*. *oblique*-inducible miRNAs target transcription factors such as csn-miR156/SPL, csn-miR160/ARF, csn-miR164/NAC, csn-miR169/NFY, csn-miR171/bHLH, csn-miR172/ERF, csn-miR319/TCP, csn-miR396/GRF and csn-miR828/MYB (Fig. 5 and Supplementary Table S5). Among them, miR156, miR164, miR171, miR172, miR319 and miR828 were also identified as being specifically induced by OS of insect herbivory^31^. Bozorov *et al*.^31^ found that miR160, miR167 and miR396 were induced by MW stress, but not by OS of insect herbivory. However, miR160 and miR167, which target *ARF10* and *ARF8*, respectively, were up-regulated in response to salt stress and were down-regulated in response to *H*. *schachtii* infection^37^. miR396 is a negative regulator of mitotic cell division via the down-regulation of GRF genes, which were found to be responsive to various abiotic stresses including high salinity, drought, cold, and many biotic stressors such as bacteria and fungi^37,49^. Interestingly, we observed that csn-miR160, csn-miR167 and csn-miR396 are also significantly induced in the GA/MW comparison, suggesting that they may play critical and versatile roles in response to MW- and OS-induced stresses in tea plant.

In *Arabidopsis*, miR156 and miR164 were induced by infection with the *TYMV p69* virus, and were also induced in transgenic *Arabidopsis* plants expressing the viral silencing P1/HC-Pro^50^. Similarly, miR156, miR160 and miR164 were induced after viral infection in tabacco^51^. It is well-described that miR156 mainly targets SBP-box genes and miR164 mainly targets NAC genes; SBP-box genes and NAC genes were found to be involved in various biotic and abiotic stresses^52–54^. There is also evidence that miR156 and miR172 mediate ERF regulation under salt stress in radish^55^. We found that csn-miR156g, csn-miR156f, and csn-miR172g-5p targeted ERFs, which may activate defense-related genes through the ethylene pathway^56^ (Fig. 5 and Supplementary Table S5).

TCP transcription factors are the main targets of miR319, which positively regulate jasmonate (JA) biosynthesis^57^. miR319 is one of the best-studied miRNAs, which is involved in plant leaf development^57,58^, and abiotic stresses^59–61^. Remarkably, a previous study demonstrated that the miR319/TCP4 module affected JA synthetic genes and the endogenous JA level in leaves, thereby mediating root-knot nematode (RKN) resistance in tomato^62^. Our results indicated that csn-miR319c was significantly down-regulated under both GA and MW stresses based on HTS and qRT-PCR, suggesting that this miRNA may play an important role in the fine-tuning regulation of JA biosynthesis when the tea plant is attacked by herbivory^63^. Thus, csn-miR156, csn-miR172 and csn-miR319 might be involved in cross-talk among biotic stress pathways related to *C*. *sinensis*.

MYB is a well-described and large family of transcription factors in plants, which are involved in the regulation of a wide range of molecular events such as cell cycle, hormone regulation, and stress-related responses^64^. In *N*. *attenuata*, a JA-induced MYB transcription factor, *NaMYB8*, is involved in plant defense against herbivory and phenylpropanoid biosynthesis^65,66^. In addition, Bozorov *et al*.^31^ found that Nat-miR828 targeting of MYB was increased by both MW and GA treatments. Nevertheless, our results showed that csn-miR828a (which also targets a MYB transcription factor) was decreased significantly in the GA/MW comparison (Fig. 5). Thus, different members of the miR828 family may be identified in *N*. *attenuata* and in tea, but this differentiation needs to be further elucidated. bHLH is another large transcription factor family in plants, which play versatile roles in plant development and especially in plant-defense responses. The interaction between MYB and bHLH controls multiple enzymatic steps in the flavonoid (anthocyanin and proanthocyanidin) biosynthesis pathway, phytochrome A (phyA) signaling, and activation of abscisic acid (ABA)-inducible gene expression; it also regulates defense-related secondary metabolite production, such as glucosinolate biosynthesis^67^. In this study, 10 miRNAs targeting bHLH genes were identified, including one conserved (csn-miR171q) and 7 novel miRNAs (up-regulated), and two novel down-regulated miRNAs (Supplementary Table S5 and Fig. 5). These miRNAs may regulate the combinatorial interaction between MYBs and bHLHs, and thereby regulate defense-related secondary metabolite production in tea plant. Furthermore, other conserved miRNAs and a large number of novel miRNAs were also differentially expressed in the GA/MW comparison (Supplementary Table S5 and Fig. 5). Although a detailed description on the expression patterns and their potential functions of these miRNAs is lacking, they are likely to play critical roles in response to herbivory attack, and it is of interest to elucidate their potential functions.

## Conclusion

A large number of small regulatory RNAs were characterized as both conserved and novel miRNAs; many target genes of these miRNAs were predicted and functionally annotated. These genes were mainly from plant-specific transcription factor families with important functions in plant development and stress responses. Identical and differential miRNAs associated with mechanical and *E*. *oblique*-induced stress were identified, along with their target genes, which may play significant roles in the regulation of stress responses. A hypothetical model of OS-induced miRNA regulatory pathways and their target genes in tea plant were derived from the data and illustrated schematically (Fig. 5). The identification and characterization of these miRNAs will help uncover the molecular mechanisms of stress resistance to herbivores in tea plant.

## Methods

### Plant growth conditions and stress treatments

The tea plant samples (*Camellia sinensis* L. var. Shuchazao) were collected from the tea plantation at Anhui Agricultural University, Hefei, China. Clone cuttings from 3-year-old tea plants were cultured in pots (30-cm diameter, 35-cm height) and grown in a green house maintained at 23 ± 3 °C with 65 ± 5% room humidity and a 16/8 h (day/light) photoperiod. The experimental plants were irrigated once per day, fertilized once per month, and covered with gauze to prevent interference from other insects. Healthy Shuchazao cuttings with uniform growth (25−30 cm in height) were used for our experiments.

For the insect feeding treatments (GA), tea geometrids (*E*. *oblique*) in the 3^rd^ larval stage were starved for 8 h, and then 20 individuals were placed onto each tea plant (on a leaf at the same position on each plant); *E*. *oblique* were removed from the tea plant after one third of the leaves were consumed^68^. For mechanical wounding (MW), tea leaves were damaged with autoclaved scissors to remove a similar amount of leaf tissue at a similar position as in the insect-feeding treatment. Leaves from the non-treated tea plants (CK) were used as controls. All treated and control leaves were collected at 3, 6, 9, 12 and 24 h after treatments; they were immediately frozen in liquid nitrogen and then stored at −80 °C for further use.

### sRNA library construction and sequencing

Total RNA was extracted from the leaves of CK, GA and MW tea plants using a total RNA purification kit (NorgenBiotek Corporation, Canada) according to the manufacturer’s instructions. The quantity and purity of the total RNA were determined with a Bioanalyzer 2100 (Agilent, CA, USA) and the RNA 6000 Nano LabChip Kit (Agilent, CA, USA) with RIN number >7.0. Three small RNA libraries were constructed according to previously reported procedures^69,70^. Briefly, small RNA fragments ranging from 18 to 30 nt in length were excised and purified from the gel, and subsequently ligated to Solexa adaptors at each end by T4 RNA ligase. Afterwards, the adapter-ligated small RNAs were reverse transcribed to cDNA with Super Script II Reverse Transcriptase (Invitrogen), and the sRNA libraries were then sequenced with the Solexa sequencing technology using an Illumina GAIIX system provided by LC Sciences (Houston, TX, USA). The generated sRNA library sequences have been deposited in the NCBI Gene Expression Omnibus (GEO, accession number: GSE98474).

### Bioinformatics analysis of the sequencing data

The raw read sequences obtained from the Illumina GAIIX system were processed according to the procedures described in a previous study^71^ by LC Sciences. In brief, the raw reads were filtered using the Illumina pipeline filter (Solexa 0.3), and then further processed with an in-house program, ACGT101-v4.2-miR (LC Sciences, Houston, Texas, USA), to remove adapter dimers, junk, low complexity reads, common RNA families (rRNA, tRNA, snRNA, snoRNA) and repeats. Unique sequences ranging from 18 to 26 nt in length were mapped with *C*. *sinensis* reference sequences, including tea genome scaffolds and contigs from whole-genome shotgun sequencing and assembly, tea transcriptome sequences from the NCBI Sequence Read Archive (SRA, GenBank accession no. SRR1979118), EST sequences, genomic survey sequences (GSS’s) and nucleotide sequences downloaded from the GenBank nucleotide databases at NCBI (http://www. ncbi.nlm.nih.gov). The mapped sequences were aligned against known plant miRNAs from a publicly available database (http://www.mirbase.org, release 21)^17^, and BLAST searches were used to identify known miRNAs with no more than two mismatches. The sequencing reads failing to match any known miRNAs in miRBase were further analyzed to identify any novel miRNAs based on the characteristic hairpin structures of miRNA precursors according to the criteria for novel miRNAs^72^. The stem-loop secondary structures of candidate miRNA precursors were predicted using the RNAfold program as previously described by Zuker^29^. The expression abundance of miRNAs (conserved and novel) in the three libraries was normalized to one million using the following normalization formula: normalized expression = original counts of miRNA/total count of clean reads ×1,000,000. The fold changes in stress-regulated miRNAs were calculated as the log_2_ values (GA or MW normalized reads/control normalized reads), as reported earlier^73^. Positive log_2_ (≥2) and negative log_2_ (<−0.5) values at *P* ≤ 0.05 were used as the thresholds to identify up-regulated and down-regulated miRNAs, respectively, under GA and MW stress^74^.

### miRNA microarray analysis

The miRNA microarray analysis was performed by LC Sciences, Houston, Texas, USA. Briefly, total RNA (5 µg) extracted from the GA, MW and CK plants was used as the starting material for microarray hybridization (three biological replicates were used). RNAs were size-fractionated using the YM-100 Microcon centrifugal filter (Millipore, Bedford, MA, USA). The fractionated sRNAs (<30 nt) were extended with a poly (A) tail at the 3′ end using poly (A) polymerase and ligated to an oligonucleotide tag for subsequent fluorescent dye staining. A total of 642 probes representing all the predicted miRNAs from high-throughput sequencing were spotted onto each chip. Each chip was manufactured using three replicates per miRNA probe. The miRNA probe sequence consisted of a chemically modified nucleotide code designed to be fully complementary to the cognate target miRNA. Chip hybridization was performed using a microcirculation pump (Atactic Technologies). The hybridization melting temperature of 34 °C was made uniform by chemical modifications of the detection probes. The hybridization experiment was performed using 100 μL of hybridization buffer containing 6× SSPE (0.90 M NaCl, 60 mM Na_2_HPO_4_ and 6 mM EDTA [pH 6.8]) and 25% formamide. Hybridized arrays were read with a laser scanner (GenePix 4000B; Molecular Device, Sunnyvale, CA, USA), and the images were digitized with the Array-Pro image analysis software (Media Cybernetics, Silver Spring, MD, USA). During data analysis, the background was subtracted and signal normalization was performed using the LOWESS program. Spots of intensity that were less than three-fold that of the background standard deviation, and with a spot coefficient of variation (CV) greater than 0.5 were removed. CV was calculated as the standard deviation/signal intensity. To minimize noise and to improve accuracy, some probes with low abundances (signal values < 100) were not included in the variance analysis. Signal values below the background average (<32) were considered to indicate non-expression.

### Target gene prediction

To predict the potential target genes of miRNAs, we used Target Finder resources (http://www.danforthcenter.org/scientists-research/principal-investigators/james-carrington/) against the transcriptome sequence data of *C*. *sinensis* deposited in the NCBI Sequencing Read Archive database under accession number SRR1979118. The predicted target genes were evaluated based on the complementarity scores and maximum expectation according to Bo and Wang^75^.

### GO and KEGG analysis

To better understand the function of the target genes and their corresponding metabolic networks during GA and MW stress in tea plant, functional annotations of each target gene was performed using the GO and KEGG pathway database. The predicted targets were annotated based on sequence similarity by performing a BLASTX search against the GO protein database. Furthermore, target genes were categorized according to function under biological processes, molecular functions and cellular components using GO analysis. The enriched metabolic pathways or signal transduction pathways of potential miRNA target genes were validated using KEGG enrichment analysis to enrich the KEGG terms according to Zhang *et al*.^69^.

### Validation of miRNAs using stem-loop qRT-PCR

To validate the predicted mature known and novel miRNAs in this study, we used stem-loop qRT-PCR^76^. The stem-loop RT primers consisted of 44 conserved and 6 variable nucleotides that are specific to the 3′-end of the miRNA sequences (5′-GTC GTA TCC AGT GCA GGG TCC GAG GTA TTC GCA CTG GAT ACG ACN NNN NN-3′). Forward primers, composed of six nucleotides at the 3′-end of the stem-loop RT primer that were complementary to the 3′-end of the miRNA, were designed for each individual miRNA according to Varkonyi-Gasic *et al*.^77^, and 5′-GTG CAG GGT CCG AGG TAT TC-3′ was used as the reverse primer. U6 (a small noncoding RNA) was used as an internal control. Detailed information about the primers used in this study is presented in Supplementary Table S6.

Total RNA was isolated from tea leaves subjected to MW, treated with the initial concentration of *E*. *oblique* oral secretion (OS) and 5× diluted concentration of OS (OS-5×); isolation was performed with the total RNA purification kit (NorgenBiotek Corporation, Canada) according to the manufacturer’s protocol. cDNA was synthesized in a volume of 20 μL containing 500 ng of total RNA, 4 μL 5× PrimeScript buffer, 0.5 μL M-MLV reverse transcriptase (Takara, Dalian, China), and 1 μL stem-loop RT primer (1 μM). After pre-denaturation at 65 °C for 5 min, the mixture was incubated on ice for 2 min, and then, the RT reaction was performed for 30 min at 16 °C, followed by 60 cycles of 30 °C for 30 s, 42 °C for 30 s and 50 °C for 1 s, and a final hold at 85 °C for 5 min.

qRT-PCR was performed on the CFX96 real time detection system (Bio-Rad, Hercules, USA) using a SYBR Premix Ex TaqTMII kit (Takara, Dalian, China). The final volume was 25 μL, and it contained 2 μL cDNA, 8.5 μL ddH_2_O, 12.5 μL SYBR Premix Ex TaqTMII, 1 μL specific forward primer (10 μM) and 1 μL universal reverse primer (10 μM). The reaction was incubated at 95 °C for 5 min, followed by 40 cycles of 95 °C for 5 s and 60 °C for 10 s. U6 was used as an internal reference. The same reaction conditions were used for the no-template controls and RT-minus controls. All reactions were performed in three technical replicates. The relative expression of miRNA for validation of the predicted miRNAs was calculated using the 2^−ΔΔCt^ method^78^. Three biological replicates of all samples were analyzed.

### Data availability

The sequences from the small RNA library have been deposited in the Gene Expression Omnibus (GEO) database, the accession number is GSE98474 (https://www.ncbi.nlm.nih.gov/geo/query/acc.cgi?acc=GSE98474). The transcriptome sequence data of *C*. *sinensis* was deposited in the NCBI SRA database with accession SRR1979118.

## Electronic supplementary material

Dataset 1 Table S1.Summary of conserved miRNAs from small RNA libraries

Dataset 2 Table S2. Summary of novel miRNAs from small RNA libraries

Dataset 3 Table S3. Expression patterns of conserved and novel miRNAs identified in CK-Vs-GA

Dataset 4 Table S4. Expression patterns of conserved and novel miRNAs identified in CK-Vs-MW

Dataset 5 Table S5. Potential target transcripts of differentially expressed conserved and novel miRNAs in Camellia sinensis

Dataset 6 Table S6. List of tea miRNAs and primer sequences used for stem loop qRT-PCR analysis
